# Organic fertilizer substitution optimizes aroma metabolites in Wuyi Rock tea

**DOI:** 10.3389/fpls.2025.1581120

**Published:** 2025-06-18

**Authors:** Shuping Huang, Yongdong Yu, Jilai Cui, Zhengwei Luo, Lanxin Luo, Chuankui Song, Hong Liao

**Affiliations:** ^1^ Root Biology Center, College of Resources and Environment, Fujian Agriculture and Forestry University, Fuzhou, China; ^2^ Dabie Mountain Laboratory, College of Tea and Food Science, Xinyang Normal University, Xinyang, Henan, China; ^3^ Henan Key Laboratory of Tea Plant Biology, College of Tea and Food Science, Xinyang Normal University, Xinyang, Henan, China; ^4^ State Key Laboratory of Tea Plant Biology and Utilization, Anhui Agricultural University, Hefei, Anhui, China

**Keywords:** organic fertilizer, aroma, soil, Wuyi Rock tea, metabolites

## Abstract

Pleasant aroma is a distinctive character of Wuyi Rock tea, but its optimization through agricultural practices remains largely unexplored. Here, we conducted a two-year field trials in the core-region of Wuyi Rock tea production area with organic or chemical fertilizer. The results indicated that organic fertilizer significantly improves soil fertility, as indicated by increased pH and organic matter. GC-MS analysis showed that organic fertilizer obviously affects the aroma metabolites in tea leaves, with the levels of 1-Hexanol (C_6_H_14_O), 2-Ethyl-1-hexanol (C_8_H_18_O), (E,E)-2,4-Heptadienal (C_7_H_10_O), E-Nerolidol (C_15_H_26_O) and 3-octen-2-one (C_8_H_14_O) increasing by 56.55%, 104.44%, 64.39%, 64.10% and 48.10%, respectively, compared to chemical fertilizer, thereby improving its aroma quality. The correlation analysis and PLS-PM model combined with the results from ionomics and metabolomics, further elucidated that soil fertility significantly impacted the mineral nutrients in tea leaves, thereby regulating the content of volatile metabolites. Altogether, the research findings provide practical fertilizer usage guidelines for tea farmers, helping to improve the aroma quality and overall market value of tea.

## Introduction

Oolong tea is renowned for its exquisite processing, time-honored history, health benefits, and unique taste (Wang et al., 2022a). Among the various types of oolong tea, Wuyi Rock tea, produced in Wuyi Mountain City of Fujian, is a paragon among Fujian oolong teas. Wuyi Rock tea is globally recognized for its unique rock rhyme, rich texture, and aroma, securing its niche in the tea industry (Ho et al., 2015). The characteristic flavor of Wuyi Rock tea primarily stems from its distinctive manufacturing process, which involves withering, tumbling and aeration, enzyme inactivation, shaping, and drying, bestowing the tea with its unique taste and floral aroma (Liu et al., 2022a). While many studies have focused on identifying aromatic compounds in the production process and the final product (Zheng et al., 2022), the influence of fresh tea leaves on Wuyi Rock tea’s aroma is still poorly understood.

Fertilization is a crucial management practice that ensures both tea yield and quality. Nutrient deficiency can notably reduce the content of amino acids and aromatic compounds, with the L-theanine content decreasing to 11.4% of the control level (Zhou et al., 2022), thereby degrading the quality of tea. While chemical fertilizers are widely used to ensure nutrient supply, their long-term excessive and improper use can reduce the biosynthesis of polyphenols and flavonoids, leading to a bitter taste in tea (Ye et al., 2022). This practice also threatens the sustainable production of tea (Arafat et al., 2019; Ni et al., 2019). High-phosphorus conditions decrease the accumulation of polyphenols in tea plants (Zhang et al., 2023), while elevated phosphorus and potassium reduce free amino acids such as theanine and glutamic acid, simultaneously increasing flavonoid-related metabolites (Wei et al., 2022). Moreover, overuse of chemical fertilizers leads to soil degradation, including decreased beneficial microorganisms, nutrient leaching, soil structure deterioration, and acidification (Li et al., 2016).

In contrast to chemical fertilizers, organic fertilizers offer several benefits for soil health and tea quality. Organic fertilizer can effectively alleviate soil acidification and increase soil organic matter (Wang et al., 2025). Studies indicate that the rational use of organic fertilizers can promote the synthesis of amino acids and flavonoids in tea (Ruan et al., 2019). However, relying solely on organic fertilizers can reduce tea yield by 10-20%, impacting economic profitability (Das et al., 2016; Piyasena and Hettiarachchi, 2023). Currently, many tea plantations adopt a fertilization strategy that integrates organic and chemical fertilizers. This approach ensures tea yield (Manzoor et al., 2024), promotes catechin and flavonoid synthesis in tea leaves (Raza et al., 2024) and increases the levels of aromatic compounds such as D-limonene, linalool, and cis-3-hexenyl hexanoate in green tea (Huang et al., 2022). Nevertheless, more research is needed to understand the effects of using organic fertilizers alone on tea plantation soils and tea quality.

It was reported that a high-nitrogen, low-phosphorus, and medium-potassium nutrient ratio is optimal for the growth of high-quality Wuyi Rock tea (Wang et al., 2022b). Based on this, we developed a tea plant-specific organic fertilizer. To explore the effects of organic fertilizer on the yield and quality of Wuyi Rock tea, we established a two-year field trial including organic fertilizer and chemical fertilizer. The impact of different fertilization treatments on the soil of tea plantations and the key metabolites of tea plants was analyzed using Inductively Coupled Plasma Mass Spectrometry (ICP-MS) and Gas Chromatography-Mass Spectrometry (GC-MS). Further, our study elucidated the effects of organic fertilizer on the yield and quality of Wuyi Rock tea, providing a theoretical reference for rational fertilization and the enhancement of tea quality in Wuyi tea plantations, while offering practical fertilizer usage guidelines for tea farmers to improve the aroma quality and overall market value of tea.

## Materials and methods

### Plant material and fertilizer treatment

The tea variety (*Camellia sinensis* L. cv. “Rougui”) was used in this study. The trial field of tea plants was located in the core-region of Wuyi Rock tea production area, Wuyi Mountain city, Fujian province, China (27°32’36”-27°55’15”N; 117°24’12”-118°02’50”E). The basic soil chemical properties of the soil (0–20 cm) were as follows: pH 4.25; alkali-hydrolysable nitrogen (AN), 77.68 mg/kg; available phosphorus (AP), 145.68 mg/kg; rapid-acting potassium (AK), 143.75 mg/kg; and organic matter (OM), 19.55 mg/kg.

This experiment employed two fertilization treatments: chemical fertilizers and organic fertilizer. Each treatment was replicated three times, with plot sizes of 60 m² (6 m × 10 m), ensuring a consistent spacing of 1.5 m between rows of tea trees. The application rate for both fertilization treatments was uniformly set at 750 kg/ha. For the chemical fertilizer treatment, a compound fertilizer was selected, with a nutrient ratio of N:P_2_O_5_:K_2_O = 15:15:15. In contrast, the organic fertilizer treatment utilized a tea-specific organic fertilizer, primarily composed of cow manure, spent mushroom substrate, and soybean meal, which underwent ultra-high temperature fermentation (exceeding 80°C), resulting in a nutrient ratio of N:P_2_O_5_:K_2_O = 10:1:5, and an organic matter content of no less than 30%. The fertilization practices were conducted during the autumn seasons of 2022 and 2023, employing trench application methods, with the depth of fertilization controlled at 20 cm.

### Collection of tea leaves and soil samples

Tea leaf samples (one-tip-three-leaf) were manually harvested in May 2023 and May 2024. Fresh leaf samples, amounting to 20 grams from each tea plant, were collected, with 10 plants chosen at random for biological replication. The samples were fixed at 105°C for 30 minutes, then dried at 75°C until reaching a constant weight, and subsequently ground for further analysis. Soil samples were gathered from 10 random sites within each plot in July 2023 and July 2024, after visible plant residues and stones were removed, using a stainless steel soil corer to collect samples from a depth of 0–20 cm. The soil samples were air-dried, pulverized, and sieved through 2 mm and 0.0149 mm screens for subsequent analysis.

### Analysis of nutrient concentrations

The mineral elements in tea leaf were measured according to methods previously described (Peng et al., 2018). In brief, the concentration of macronutrients (N, P, K) was measured by a flow autoanalyzer (SKALAR SAN++, Skalar, Breda, Netherlands). The concentrations of calcium (Ca), Natrium (Na), magnesium (Mg), aluminum (Al), manganese (Mn), iron (Fe), zinc (Zn), copper (Cu), boron (B), nickel (Ni), molybdenum (Mo) and cobalt (Co) were measured by inductively coupled plasma-mass spectrometry (ICP-MS 7900, Agilent Technologies, Santa Clara, California, US).

### Analysis of physicochemical properties of soil samples

The soil samples were measured using the method as described by Liu et al. (2022b). In brief, the soil pH was determined using a pH meter (ORION3STAR, Thermo Fisher Scientific, USA) in a 1:25 (w/v) paste with deionized water. OM and AN were quantified using high-temperature potassium dichromate oxidation and the alkali-hydrolysable diffusion method, respectively. AP was extracted using the BrayI method, and readily AK was extracted from an ammonium acetate solution.

### Analysis of volatiles via GC-MS

Gas chromatography-mass spectrometry (GC-MS) was performed according to the previous study (Cui et al., 2023). The volatile compounds were examined utilizing a TRACE 1300 gas chromatograph coupled with a DSQ II mass spectrometer (Thermo Fisher Scientific, Waltham, USA). A 1 μL aliquot of the previously prepared concentrated distillate was introduced into the GC in a splitless mode at an injection port temperature of 250°C. Separation of the volatiles was achieved on a DB-5 fused silica capillary column DB-5, 30 m × 0.25 mm, 0.25 μm film thickness, J&W Scientific, CA, USA). The GC column oven temperature was programmed to initially maintain at 50°C for 5 minutes, subsequently increase at a rate of 4°C per minute to 160°C (held for 3 minutes), and further ascend at 8°C per minute to 230°C (held for 2 minutes). Helium (purity > 99.999%) served as the carrier gas, flowing at a rate of 1 mL per minute. The ion source functioned in positive ion mode at an ionization energy of 70 eV and a temperature of 230°C. The volatiles were detected using full scan mode with a mass range of m/z 35-450.

A mixture of n-alkanes (C8–C25) was injected under the same GC-MS conditions as the samples to calculate the retention index (RI) of each volatile. The volatiles were identified by comparing the detected mass spectra to those in the National Institute of Standards and Technology (NIST17) database. Compounds with a mass spectra similarity higher than 800 and an RI difference of <20 were identified as volatiles in samples. When available, compounds were also identified based on the retention times, retention indices and mass spectra of the standards. The volatile compounds were quantified by comparing the peak area of each compound to that of the internal standard (ethyl caprate) using their respective characteristic ions (**Supplementary Table 2**).

### Data analysis

All experimental data were organized using Microsoft Excel 2019 (Microsoft Corporation, USA). Data processing and figure creation were conducted with Graphpad Prism 9.5 software and R language packages. The obtained data were subjected to normality tests, two-way ANOVA, and Student’s *t*-test using the SPSS sofaware (Version 19.0.0, International Business Machines Corporation, Chicago, America). OPLS-DA analysis (Orthogonal Partial Least Squares-Discriminant Analysis), Mantel test analysis and PLS-PM analysis were performed using the “ropls,” “linkET,” and “plspm” packages in R. *: 0.01 < *P* ≤ 0.05; **: 0.001 < *P* ≤0.01; ***: *P* ≤ 0.001, ns: no significant difference.

## Results

### The effects of organic fertilizer substitution on soil fertility

**Figure 1:**
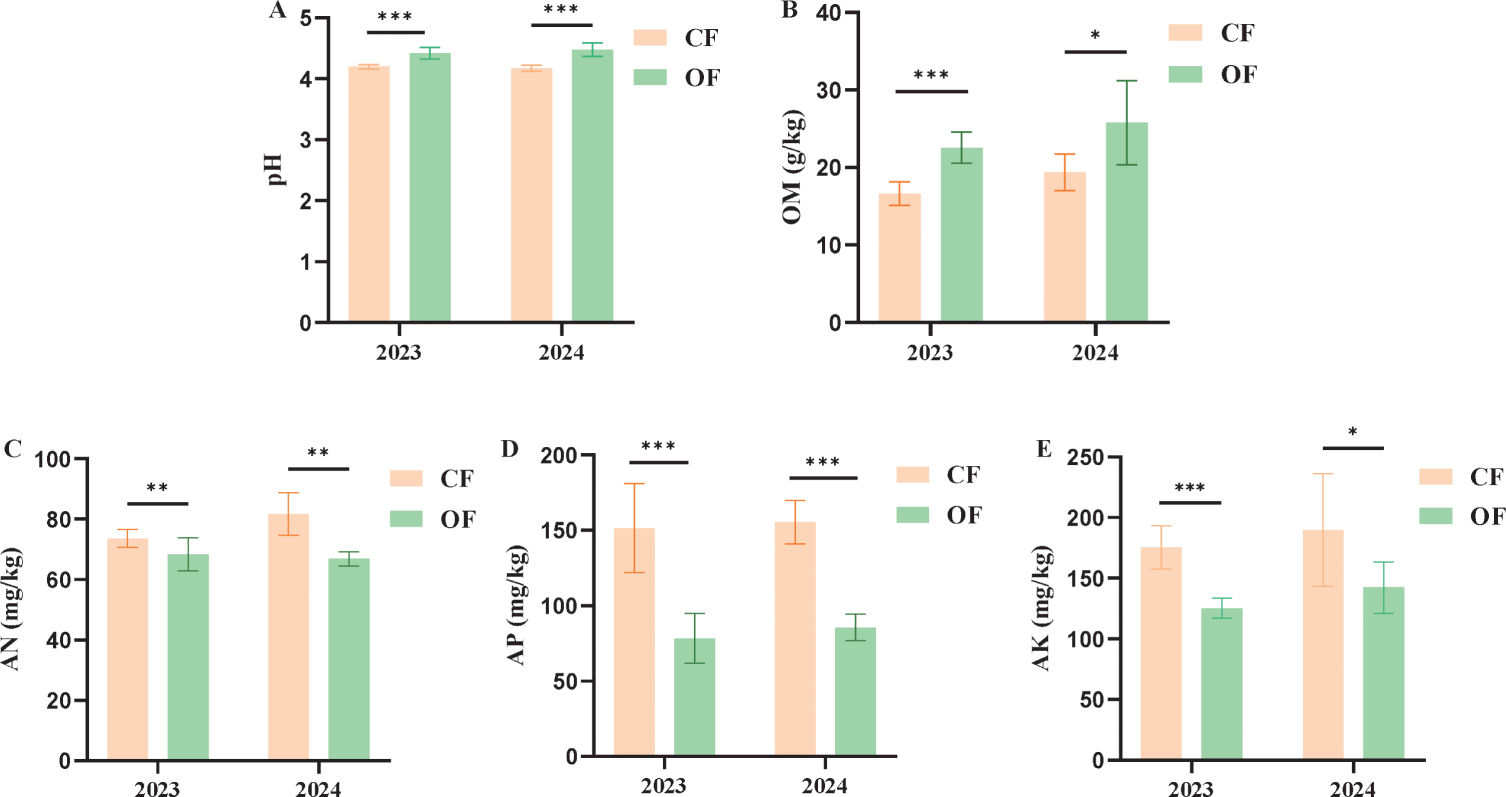
Soil fertility changes in tea plantation. **(A)** Soil pH; **(B)** Soil organic matter content; **(C)** Soil alkali-hydrolysable nitrogen content; **(D)** Soil available potassium content; **(E)** Soil available phosphorus content; n = 10. CF, chemical fertilizer; OF, organic fertilizer. The differences between groups were assessed using *t*-tests. *0.01 < *P* ≤ 0.05; **0.001 < *P* ≤0.01; ***P ≤ 0.001, ns, no significant difference.

To investigate the impact of organic fertilizer substitution on tea plantation soils, five soil fertility indexes were measured, including pH value, organic matter (OM), alkali-hydrolysable nitrogen (AN), available phosphorus (AP) and available potassium (AK) concentrations. Interestingly, the average soil pH values in the tea plantation treated with organic fertilizer for two years were 4.42 and 4.48, while treated with chemical fertilizer had average soil pH values of 4.19 and 4.17 (**Figure 1A**), showing that organic fertilizer treatment effectively mitigated soil acidification. The soil OM content was also significantly increased, with increases of 35.66% and 33.02% compared to the chemical fertilizer treatment (**Figure 1B**). In contrast, after two years of organic fertilizer application, the soil’s AN, AP and AK in the tea plantation decreased by 7.07% and 18.22%, 18.28% and 44.93%, and 28.66% and 25.03% (**Figures 1C-E**), respectively, compared to the chemical fertilizer application. These results indicated that organic fertilizer significantly changes soil fertility in tea plantation.

### The effects of organic fertilizer substitution on tea quality

A two-year field trials in the core-region of Wuyi Rock tea production area was conducted to study the effects of organic fertilizer on tea yield and quality. Results showed that organic fertilizer application did not decrease tea yield but increased the number of standard leaves per unit area compared to chemical fertilizer treatment (**Supplementary Figure 1**). Analysis of mineral nutrients revealed that organic fertilizer significantly reduced phosphorus (P), magnesium (Mg), iron (Fe), and zinc (Zn) concentrations but increased boron (B) concentration in tea leaves relative to chemical fertilizer (**Table 1**).

**Table 1:** Table of mineral element concentrations in fresh tea leaves under various fertilization treatments.

Mineral Nutrient	Treatments		*P*
	OF	CF	
N (mg·g^-1^)	28.01 ± 1.30	28.75 ± 1.46	0.255 ns
P (mg·g^-1^)	2.74 ± 0.17	2.94 ± 0.14	0.015^*^
K (mg·g^-1^)	12.38 ± 0.62	11.95 ± 0.47	0.094 ns
Ca (mg·g^-1^)	3.34 ± 0.46	3.45 ± 0.33	0.556 ns
Mg (mg·g^-1^)	1.58 ± 0.19	1.82 ± 0.18	0.011^*^
Al (mg·g^-1^)	0.64 ± 0.08	0.59 ± 0.06	0.201 ns
Mn (μg·g^-1^)	105.78 ± 25.97	112.01 ± 21.1	0.563 ns
B (μg·g^-1^)	26.44 ± 6.46	19.86 ± 4.40	0.016^*^
Na (μg·g^-1^)	30.61 ± 15.67	22.08 ± 6.84	0.132 ns
Fe (μg·g^-1^)	62.22 ± 4.85	80.11 ± 10.11	0.0002^***^
Co (μg·g^-1^)	0.07 ± 0.03	0.09 ± 0.03	0.689 ns
Ni (μg·g^-1^)	4.42 ± 0.96	4.87 ± 0.84	0.281 ns
Cu (μg·g^-1^)	6.62 ± 0.9	7.17 ± 1.02	0.221 ns
Zn (μg·g^-1^)	9.39 ± 2.83	12.31 ± 2.34	0.022^*^
Mo (μg·g^-1^)	0.06 ± 0.03	0.09 ± 0.25	0.265 ns

The differences between groups were assessed using t-tests. * 0.01<P≤0.05; ***P≤0.001, ns, no significant difference.

**Figure 2:**
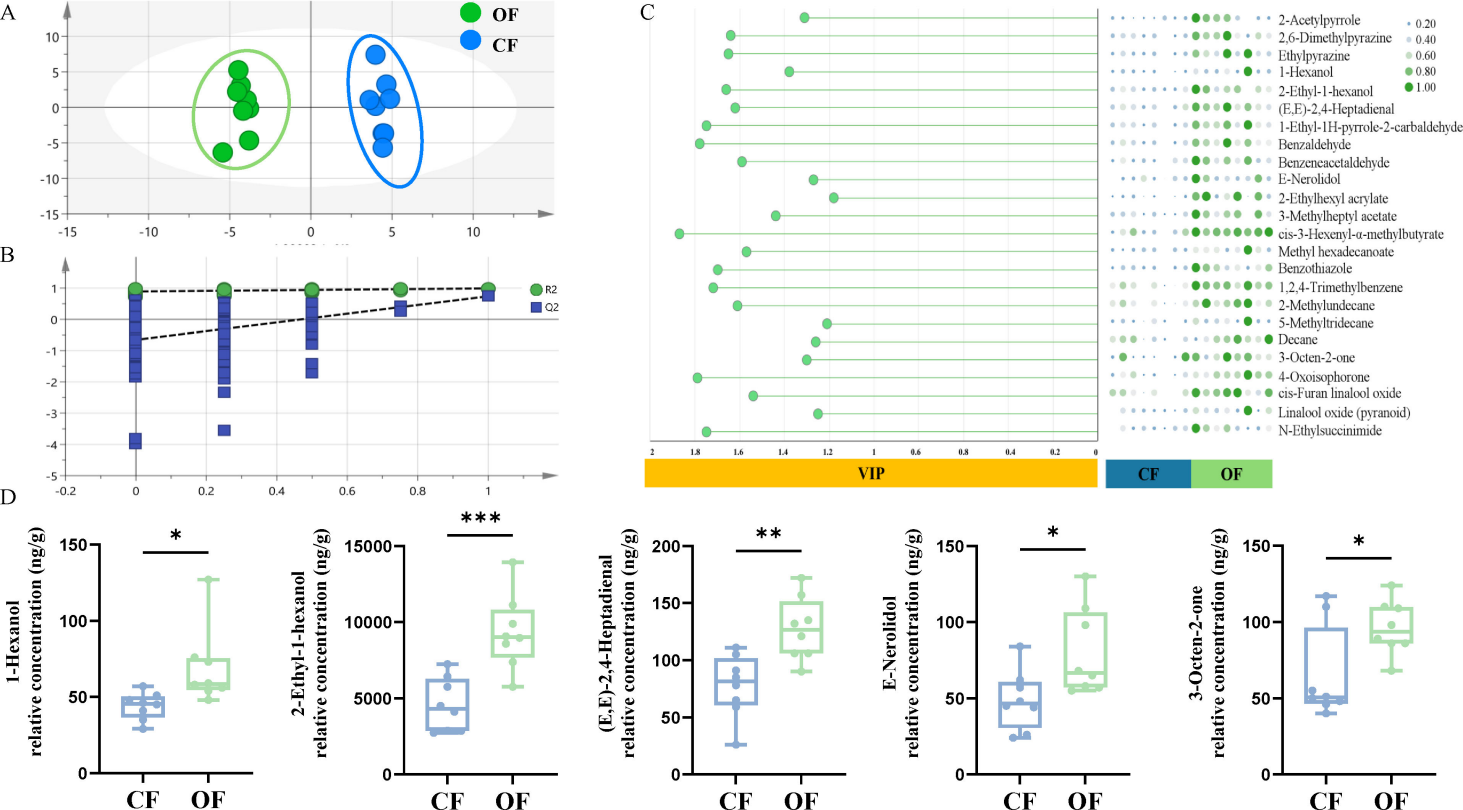
Differential metabolite processing analysis chart. **(A)** OPLS-DA model plot; **(B)** Cross-validation plot of the PLS-DA model; **(C)** VIP values and heatmap of differential metabolites **(D)**; Box plot of differential aroma metabolites. The differences between groups were assessed using *t*-tests. *0.01 < *P* ≤ 0.05; **0.001 < *P* ≤0.01; ***P ≤ 0.001, ns, no significant difference.

To investigate the impact of organic fertilizer on the volatile metabolites of tea leaves, an analysis of volatile metabolites was conducted. A total of 113 volatile metabolites in tea leaves were identified through GC-MS analysis (**Supplementary Table 1**). An OPLS-DA model (**Figure 2A**) with high reliability (R2x = 0.651, R2y = 0.966, Q2 = 0.557, **Figure 2B**) revealed 25 differential metabolites (6 alcohols, 5 aldehydes, 4 esters, 2 ketones) based on VIP > 1 and *P* < 0.05 (**Figure 2C**). The content of key aroma compounds—1-hexanol, 2-Ethyl-1-hexanol, (E,E)-2,4-Heptadienal, E-Nerolidol, and 3-Octen-2-one—increased by 56.55%, 104.44%, 64.39%, 64.10%, and 48.10%, respectively, under organic fertilizer treatment (**Figure 2D**). These findings indicate that organic fertilizer enhances tea aroma quality by increasing aldehyde, alcohol, and ester metabolites without compromising yield.

### The relationship between mineral nutrients and aroma metabolites

**Figure 3:**
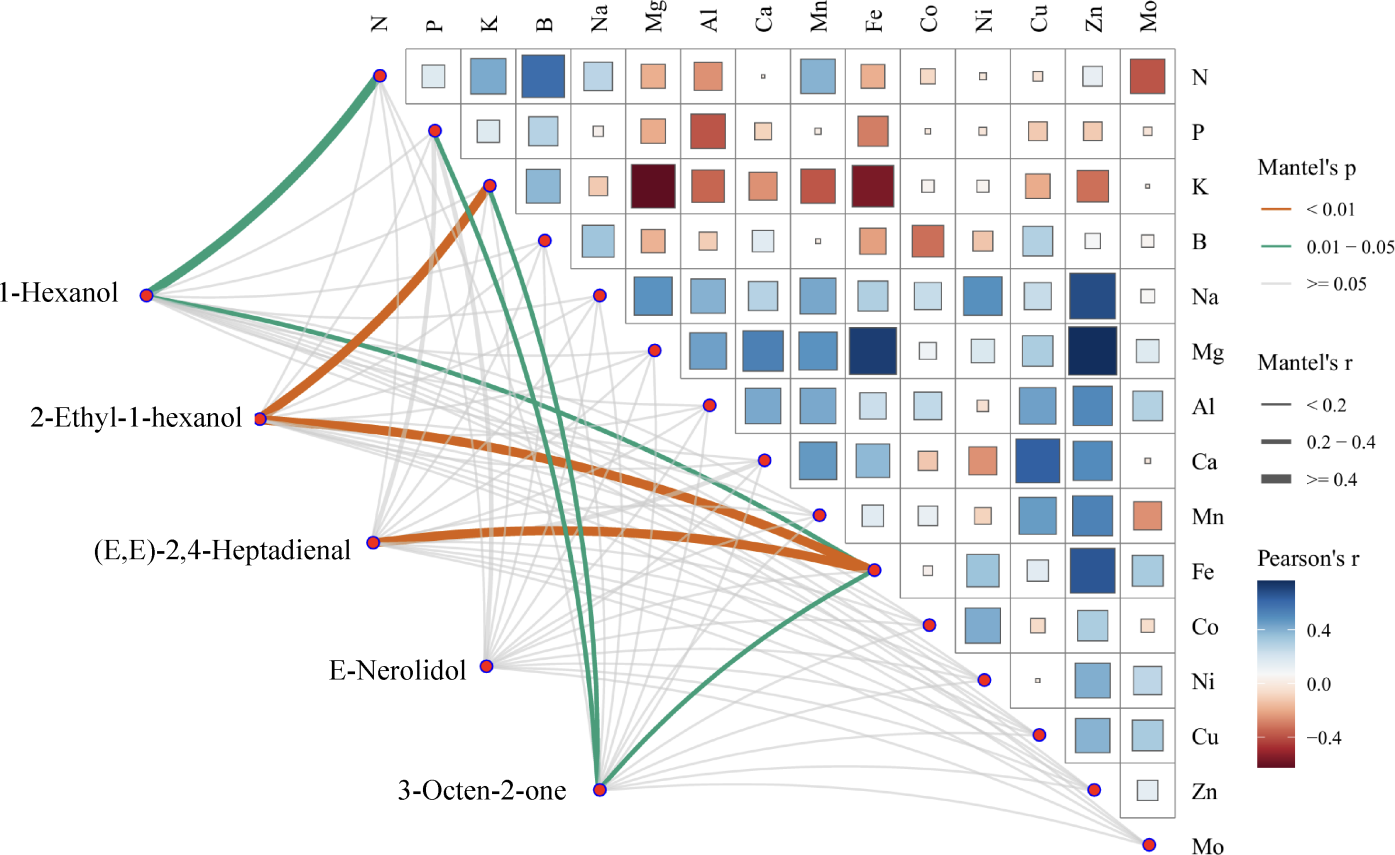
Correlation network between mineral nutrients and 1-hexanol, 2-Ethyl-1-hexanol, (E, E)-2,4-Heptadienal, E-Nerolidol and 3-Octen-2-one. The color scale represents Pearson’s correlation coefficients: blue for positive correlations, red for negative correlations. Mantel’s *P*-values are indicated by light (0.01 to 0.05) and green (> 0.05) shading, with line thickness corresponding to the strength of Mantel’s correlation (r), where thicker lines denote stronger correlations.

To explore the impact of mineral nutrients on the volatile metabolites in fresh tea leaves, mantel test correlation analysis was conducted between 1-Hexanol, 2-Ethyl-1-hexanol, (E,E)-2,4-Heptadienal, E-Nerolidol, 3-Octen-2-one and various mineral nutrients (**Figure 3**). The result revealed that 1-Hexanol exhibited correlations with N and Fe. (E,E)-2,4-Heptadienal demonstrated correlations with Fe. Furthermore, 3-Octen-2-one exhibited correlations with P, K and Fe. Notably, 2-Ethyl-1-hexanol showed a significant correlation with K and Fe. Collectively, these results indicate that mineral nutrients were significantly associated with key tea aroma metabolites.

### The relationship between soil fertility and aroma metabolites

**Figure 4:**
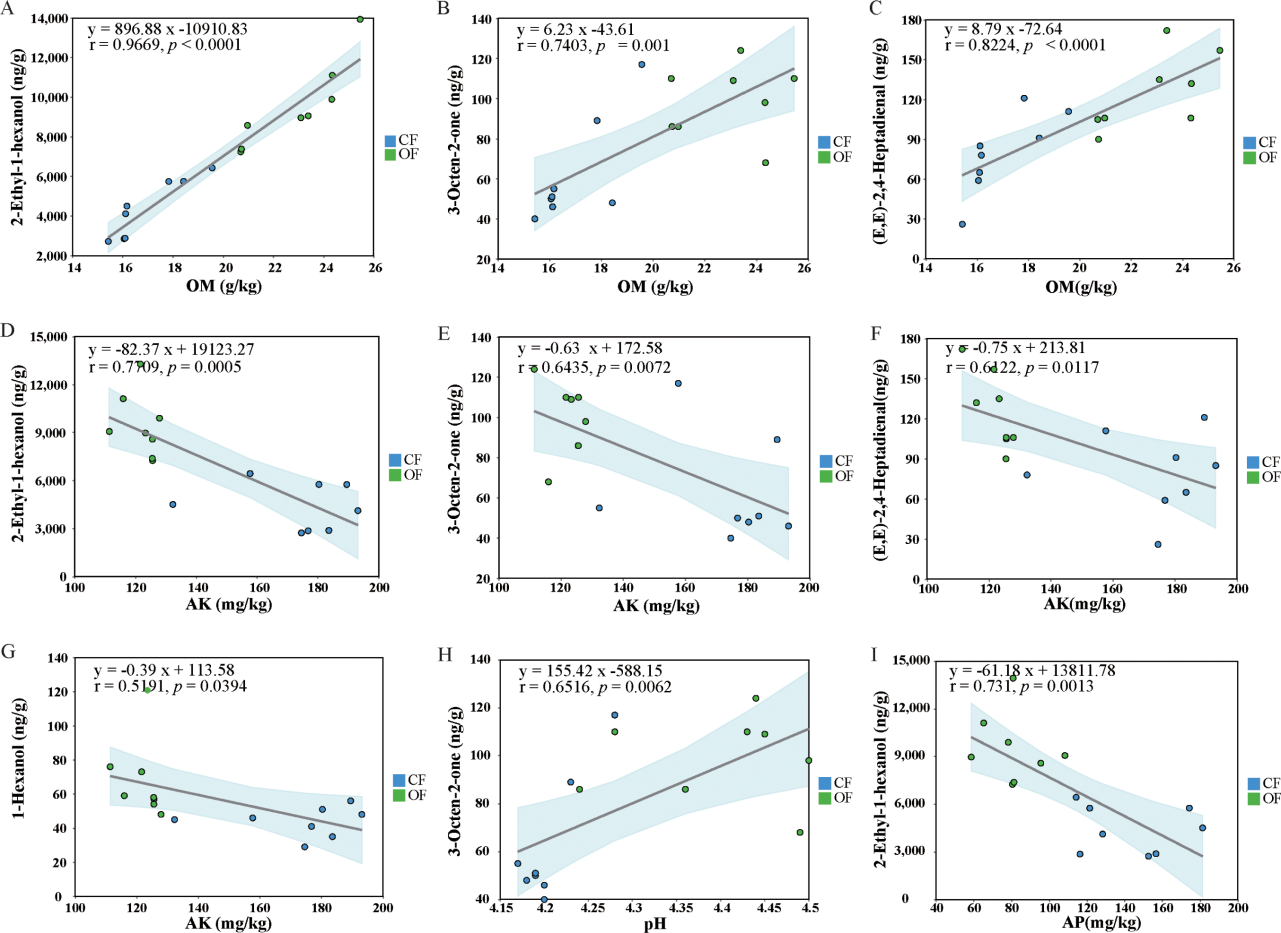
The correlation diagram of soil nutrients and differential aroma metabolites **(A)** Linear regression plot of 2-Ethyl-1-hexanol versus OM; **(B)** Linear regression plot of 3-Octen-2-one versus OM; **(C)** Linear regression plot of (E,E)-2,4-Heptadienal versus OM; **(D)** Linear regression plot of 2-Ethyl-1-hexanol versus AK; **(E)** Linear regression plot of 3-Octen-2-one versus AK; **(F)** Linear regression plot of (E,E)-2,4-Heptadienal versus OM; **(G)** Linear regression plot of 1-Hexanol versus AK; **(H)** Linear regression plot of 3-Octen-2-one versus available pH; **(I)** Linear regression plot of 2-Ethyl-1-hexanol versus AP. The shaded areas represent the 95% confidence intervals.

To further elucidate the influence of soil fertility on the volatile metabolites of fresh tea leaves, 1-hexanol, 2-Ethyl-1-hexanol, (E,E)-2,4-Heptadienal, E-Nerolidol and 3-Octen-2-one were selected for linear regression analysis with soil nutrients. The results showed that 2-Ethyl-1-hexanol, (E,E)-2,4-Heptadienal and 3-Octen-2-one were significantly positively correlated with OM, and notably, all compounds also exhibited a significant negative correlation with AK (**Figures 4A-F**). In addition, the concentration of 1-Hexanol was also negatively correlated with AK (**Figure 4G**). 3-Octen-2-one concentrations was significantly negatively correlated with pH (**Figure 4H**). Furthermore, the concentration of 2-Ethyl-1-hexanol was significantly negatively correlated with AP (**Figure 4I**). These results indicated that OM significantly positively correlated with 2-Ethyl-1-hexanol, (E,E)-2,4-Heptadienal and 3-Octen-2-one in tea leaves, while AP and AK show negative correlations with the volatile metabolites.

### Partial least squares path model analysis

**Figure 5:**
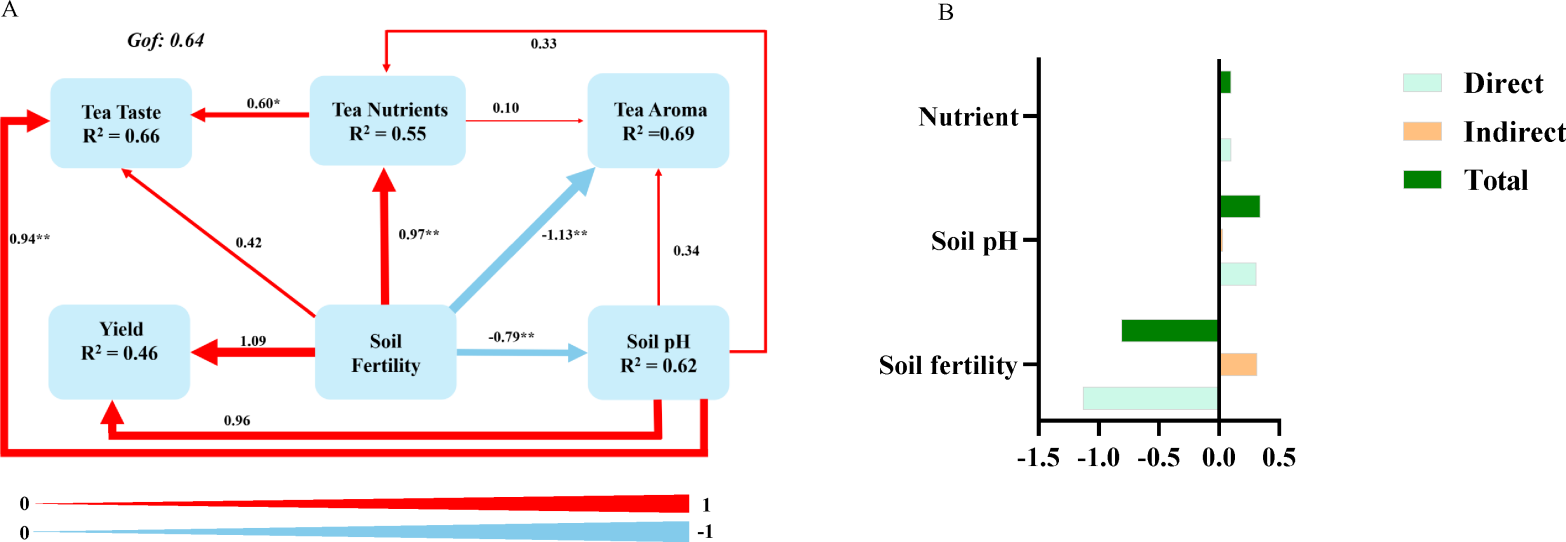
**(A)** The figures on the arrows represent standardized path coefficients, with the values of these coefficients denoted by the thickness of the arrows; red arrows signify positive impacts, while blue arrows denote negative impacts. The path coefficients and coefficients of determination (R²) are calculated following 999 bootstrap replications. The R² values indicate the variance explained by the model; the model’s evaluation is based on the Goodness of Fit statistic, a measure of overall predictive performance; pc: path coefficient, pe: path effect. **(B)** A diagram illustrating the degree of influence of tea nutrients, soil pH, and soil fertility on aroma.

To further clarify the effects of fertilization on tea yield and quality, PLS-PM model was used to reveal the relationships among soil fertility, soil pH, yield, tea nutrients, tea aroma and tea taste (**Figure 5A**). The model explained 55%, 69%, 66%, and 62% of the variation in mineral nutrients of tea, tea aroma, tea taste, and soil pH, respectively, within a goodness-of-fit index of 0.64. Soil fertility had positive direct effects on tea nutrients with a path coefficient (pc) of 0.97 and yield with a path coefficient (pc) of 1.09, whereas soil fertility exhibited a direct negative effect on tea aroma (pe = -1.13) and soil pH (pc = -0.79). Soil pH directly and significantly influenced tea yield (pc = 0.96) and tea taste (pc = 0.94), whereas contributed less to tea aroma (pc = -0.34) and tea nutrients (pc = 0.33). Additionally, the mineral nutrients of tea positively influenced tea taste (pc = 0.60) and aroma (pc = 0.10). Soil fertility exhibited the strongest total effects on tea amora, followed by soil pH and mineral nutrients of tea (**Figure 5B**). Taken together, fertilization may affect tea yield and the concentration of mineral nutrients by altering soil fertility and pH, thereby modulating the aroma of tea.

## Discussion

### The impact of organic fertilizers as a substitute for chemical fertilizers on tea yield

Harvesting annually depletes the mineral elements in tea plants, requiring the soil to be maintained rich in minerals. For tea cultivation, it is crucial to replenish the soil with sufficient mineral nutrients via fertilization (Ma et al., 2021). Effective nutrient management requires the rational use and selection of the most suitable nutrient sources (Johnston and Bruulsema, 2014). Fertilization can help restore and maintain soil nutrient levels, improving soil fertility and fostering conditions for stable, high tea yields (Ma et al., 2021). Nitrogen, phosphorus, and potassium are key macronutrients in tea plantations, but studies show that overuse of chemical fertilizers and nutrient imbalances are now urgent concerns (Mishima et al., 2010). In China, the average total nutrient input (N, P_2_O_5_, K_2_O) in tea plantations is 796 kg·hm^-2^, with about 36% receiving excessive inputs (≥ 750 kg·hm^-2^) (Ni et al., 2019). Excess nitrogen, phosphorus, and potassium can negatively impact tea quality (Chen et al., 2021). Many tea plantations have recently switched from chemical to organic fertilizers to address these issues. However, it was reported that 10-20% decrease in tea yield after the application of organic fertilizers (Das et al., 2016; Piyasena and Hettiarachchi, 2023; Shi et al., 2024). In contrast, this study found that tea plants treated with organic fertilizer saw yield increases of 14.31% and 11.11% over two years, and a rise in standard leaf counts of 17.67% and 15.51% (**Supplementary Figure 1**). These results indicate that using specialized organic fertilizer in place of chemical fertilizer can ensure the maintenance of tea yield.

### The influence of organic fertilizers substituting chemical fertilizers on tea quality

The complexity of tea flavor is largely determined by the balance of flavonoids, tea polyphenols, amino acids, and caffeine (Liu et al., 2022a). The concentration of these key compounds plays a critical role in the quality of tea (Li et al., 2022). Notably, metabolites synthesis is not only regulated by genetics, but also significantly affected by cultivation practices, fertilization strategies, and environmental factors such as temperature and light (Ahmed et al., 2019). Fertilization, as a common practice in tea plantation management, can enhance the synthesis of amino acids while reducing the ratio of tea polyphenols to total amino acids, thereby improving the overall flavor profile of tea (Qiu et al., 2024). However, different fertilizer types have varying effects on tea quality. For example, organic fertilizers typically promote amino acid and flavonoid synthesis (Ruan et al., 2019), whereas excessive chemical fertilizer use can impede polyphenol and flavonoid biosynthesis, resulting in a bitter and astringent taste (Ye et al., 2022; Piyasena and Hettiarachchi, 2023). In this study, treated with organic fertilizer, significantly increased flavonoid content in tea leaves (**Supplementary Table 1**). Additionally, the water-soluble substances in tea leaves increased significantly with organic fertilizer treatment, suggesting that specific organic fertilizers can boost the steeping durability of Wuyi Rock tea, highlighting organic fertilizers’ positive impact on tea quality.

Approximately 700 volatile compounds have been successfully identified in tea leaves, with most forming during post-harvest processing (Ho et al., 2015). Studies show that excessive nitrogen fertilizer application increases fatty acid derivatives, raising the levels of fatty acid aromatic compounds (Chen et al., 2021), negatively impacting tea quality. Conversely, moderate nitrogen fertilizer application balances lipid metabolism and aroma precursor formation, enhancing tea aroma quality (Liu et al., 2017). Aldehydes, a key component of tea aroma, are especially prevalent in green, oolong, and black teas, comprising the largest share of total volatiles (Flaig et al., 2020). Most aldehydes in tea leaves provide a citrus and green flavor profile (Zhai et al., 2022). This study found that the levels of (E,E)-2,4-Heptadienal, and E-Nerolidol significantly increased after OF treatment, enhancing tea’s fresh green aroma (**Figure 2D**). It was reported that 1-hexanol, linalool oxide, linalool, geraniol, (E)-β-ionone, isoamyl acetate, and 2-methylpropanal as contributors to a floral aroma, while 3-methyl-butanal, 2-Ethyl-1-hexanol, indole, and β-damascone were associated with a chestnut aroma (Liu et al., 2023). In this study, organic fertilizer treatment resulted in a significant increase in the levels of 1-Hexanol and 2-Ethyl-1-hexanol by 56.55% and 104.44%, respectively, thereby enhancing the aroma of tea. Furthermore, alcohol compounds are also significant volatile components in the formation of tea aroma (Zhu et al., 2021). In this study, we observed improvements in water-soluble substances, flavonoids (**Supplementary Table 1**), 1-Hexanol and 2-Ethyl-1-hexanol and other aroma components in tea leaves after organic fertilizer treatment (**Figure 2D**), strongly supporting the use of organic fertilizers to enhance Wuyi Rock tea quality.

### The effect of replacing chemical fertilizers with organic fertilizers on soil conditions

Soil, an indispensable natural resource, serves as a habitat for countless organisms and is crucial for ecological balance and human survival (Doran, 2002). The state of soil health, particularly its compositional diversity, profoundly influences crop productivity, climate stability, environmental health and human welfare (Manter et al., 2017). As a sophisticated life support system, soil contains numerous microbial communities essential to nutrient cycling (Morris and Blackwood, 2023). Nevertheless, soil health is influenced by various natural and human-induced factors (Withers et al., 2020; Yang et al., 2020) with fertilization practices having a notable effect on soil health in agricultural systems (Bai et al., 2018; Li et al., 2023). Therefore, understanding the connection between fertilization and soil health is critical for developing sustainable agricultural practices.

Tea plants are mainly grown in subtropical and tropical regions with high temperatures and heavy rainfall, leading to significant leaching of soil minerals. Numerous studies have highlighted that adding organic matter to soil greatly affects the availability of minerals and how efficiently plants absorb and use them (Sönmez et al., 2016; Turan, 2021). Nutrient use efficiency is crucial for tea growth and quality, but excess nutrients can harm tea tree growth and metabolism, potentially reducing tea quality (Chen et al., 2021). Moreover, excessive fertilization threatens the environment, potentially causing soil acidification, increased greenhouse gas emissions, and reduced biodiversity (Ma et al., 2021). Long-term reliance on inorganic fertilizers challenges sustainable land use, potentially leading to soil acidification and compaction, disrupting the nutrient balance in tea plantation soils (Yang et al., 2018). Therefore, replacing chemical fertilizers with organic fertilizers is seen as an effective way to decrease dependence on chemicals inputs. Organic fertilizers are value for their rich nutrient content, non-toxicity, environmental friendliness, long-lasting efficacy, and positive impact on soil health (Shaji et al., 2020). The average pH value of soils in Chinese tea plantations is 4.68, ranging from 3.96 to 5.48 across provinces (Yan et al., 2020). Tea trees prefer acidic soils, optimally between pH 4.0 and 5.5 (Yan et al., 2018). This study found that applying tea-specific organic fertilizer significantly raised the pH of tea plantation soil to a range of 4.28-4.54 (**Figure 1A**), benefiting tea tree growth and reducing soil acidification. This result is consistent with earlier studies (Saha et al., 2019; Zhang et al., 2019). After the organic fertilizer treatment, soil fertility in the tea plantation neared the high-quality standards for the Wuyi Tea Region (Zhou et al., 2019), providing optimal conditions for premium Wuyi Rock tea production.

The organic fertilizer enriches soil with exogenous organic matter, thus increasing organic content in the tea plantation soil (**Figure 1B**). This allows nutrients to be retained in the soil longer, in forms more accessible for plant uptake (Akinbode, 2011). It was reported that high-phosphorus conditions decrease the accumulation of polyphenols in tea plants (Zhang et al., 2023). In this study, OF treatment significantly reduced soil available phosphorus (AP) content (**Figure 1D**), which in turn decreased phosphorus levels in tea leaves (**Table 1**). Concurrently, the concentration of 2-Ethyl-1-hexanol in tea leaves increased (**Figure 2D**). The correlation analysis further revealed a significant correlation between phosphorus concentration in tea leaves and 2-Ethyl-1-hexanol content (**Figure 4I**). This suggests that organic fertilizer treatment can enhance tea aroma by reducing phosphorus levels in tea leaves, thereby increasing the concentration of aroma compounds like 2-Ethyl-1-hexanol. Consequently, the strategic use of organic fertilizer can strengthen soil quality and enhance the efficiency of nutrient uptake and use by tea plants (Birkhofer et al., 2008). In summary, this study offers theoretical and empirical evidence for combating soil acidification in tea plantations and boosting their sustainable development.

### Sustainability outlook

This study demonstrates the pivotal role of organic fertilizers in enhancing soil fertility and improving the aromatic profile of Wuyi rock tea, demonstrating that organic fertilization effectively regulates volatile metabolites and reduces soil acidification. However, it is necessary to comprehensively evaluate the long-term efficacy of organic fertilizers, extended longitudinal studies are needed to continuously monitor soil health and tea quality variations, and accumulate multi-year datasets for a more precise assessment of their enduring impact on soil and tea. Additionally, replicating experiments across diverse soil types and climatic zones is essential to validate the generalizability of the findings. These efforts will improve our understanding of organic fertilizers’ adaptability in different environmental settings and provide a strong scientific basis for broader tea cultivation practices.

## Conclusion

Tea-specific organic fertilizers, as substitutes for conventional fertilizers, maintain tea yield while significantly enhancing the levels of water-soluble substances and flavonoids, thereby improving tea taste. Moreover, the application of tea-specific organic fertilizers notably elevated the levels of key aroma compounds, including 1-hexanol, 2-Ethyl-1-hexanol, (E,E)-2,4-Heptadienal, E-Nerolidol and 3-Octen-2-one intensifying the tea’s aroma. Furthermore, the use of tea-specific organic fertilizers effectively reduced soil acidification and increased soil organic matter content in tea plantations. However, it must be noted that the present results are constrained by its regional scope, and different regions with diverse soil types and climatic conditions should be extended to enhance the generalizability of the results. In conclusion, this study provides a scientific rationale for optimized fertilization practices in Wuyi Mountain tea plantations, enhancing Wuyi Rock tea quality and the sustainability of tea garden production, while offering practical fertilizer usage guidelines for tea farmers to improve the aroma quality and overall market value of tea.

## Data Availability

The original contributions presented in the study are included in the article/**Supplementary Material**. Further inquiries can be directed to the corresponding author.

## Supplementary material

Supplementary Figure 1 Graph representing tea yield under various fertilization treatments. **(A)** Fresh tea leaf yield within an area of 0.1 m²; **(B)** Number of standard leaves within an area of 0.1 m². CF, chemical fertilizer; OF, organic fertilizer. Inter-group differences were assessed using *t*-tests; *0.01 < *P* ≤ 0.05; **0.001 < *P* ≤0.01; ****P* ≤ 0.001; ns, no significant difference; n = 10.

Supplementary Figure 2 Correlation network between soil nutrients and 1-Hexanol, 2-Ethyl-1-hexanol, (E,E)-2,4-Heptadienal, E-Nerolidol and 3-Octen-2-one.The color scale represents Pearson’s correlation coefficients: blue for positive correlations, red for negative correlations. Mantel’s *P*-values are indicated by light (0.01 to 0.05) and green (> 0.05) shading, with line thickness corresponding to the strength of Mantel’s correlation (r), where thicker lines denote stronger correlations.

Supplementary Table 1 Concentration of non-volatile metabolites in fresh tea leaves under various fertilization treatments.

Supplementary Table 2 Volatile compounds identified in fresh tea leaves.

## References

[B1] Ahmed S. Griffin T. S. Kraner D. Schaffner M. K. Sharma D. Hazel M.. (2019). Environmental factors variably impact tea secondary metabolites in the context of climate change. Front. Plant Sci. 10. doi: 10.3389/fpls.2019.00939 PMC6702324 31475018

[B2] Akinbode O. A. O. (2011). Comparative study of different organic manures and NPK fertilizer for improvement of soil chemical properties and dry matter yield of maize in t-wo different soils. J. Soil Sci. Environ. Manage. 2, 9–13. doi: 10.5897/JSSEM.9000022

[B3] Arafat Y. Tayyab M. Khan M. U. Chen T. Lin S. (2019). Long-term monoculture negatively regulates fungal community composition and abundance of tea orchards. Agronomy 9, 466. doi: 10.3390/agronomy9080466

[B4] Bai Z. G. Caspari T. Gonzalez M. R. Batjes N. H. Mäder P. Bünemann E. K.. (2018). Effects of agricultural management practices on soil quality: A review of long-term experiments for Europe and China. Agric. Ecosyst. Environ. 265, 1–7. doi: 10.1016/j.agee.2018.05.028

[B5] Birkhofer K. Bezemer T. M. Bloem J. Bonkowski M. Christensen S. Dubois D.. (2008). Long-term organic farming fosters below and aboveground biota: Implications for soil quality, biological control and productivity. Soil Biol. Biochem. 40, 2297–2308. doi: 10.1016/j.soilbio.2008.05.007

[B6] Chen Y. Z. Wang F. Wu Z. D. Jiang F. Y. Yu W. Q. Yang J.. (2021). Effects of long-term nitrogen fertilization on the formation of metabolites related to tea quality in subtropical China. Metabolites 11, 146. doi: 10.3390/metabo11030146 33801425 PMC8000315

[B7] Cui J. L. Zhou J. Du W. K. Guo D. Y. Tang X. Y. Zhao W.. (2023). Distribution of and temporal variation in volatiles in tea (*Camellia sinensis*) flowers during the opening stages. J. Agric. Food Chem. 71, 19682–19693. doi: 10.1021/acs.jafc.3c02690 37988651

[B8] Das S. Borua P. K. Bhagat R. M. (2016). Soil nitrogen and tea leaf properties in organic and conventional farming systems under humid sub-tropical conditions. Organic Agric. 6, 119–132. doi: 10.1007/s13165-015-0116-4

[B9] Doran J. W. (2002). Soil health and global sustainability: translating science into practice. Agr. Ecosyst. Environ. 88, 119–127. doi: 10.1016/S0167-8809(01)00246-8

[B10] Flaig M. Qi S. Wei G. D. Yang X. G. Schieberle P. (2020). Characterisation of the key aroma compounds in a *Longjing* green tea infusion (*Camellia sinensis*) by the sensomics approach and their quantitative changes during processing of the tea leaves. Eur. Food Res. Technol. 246, 2411–2425. doi: 10.1007/s00217-020-03584-y

[B11] Ho C. T. Zheng X. Li S. (2015). Tea aroma formation. Food Sci. Hum. Wellness 4, 9–27. doi: 10.1016/j.fshw.2015.04.001

[B12] Huang D. J. Wang Y. P. Chen X. Wu J. Wang H. J. Tan R. R.. (2022). Application of tea-specific fertilizer combined with organic fertilizer improves aroma of green tea. Horticulturae 8, 950. doi: 10.3390/horticulturae8100950

[B13] Johnston A. M. Bruulsema T. W. (2014). 4R Nutrient stewardship for improved nutrient use efficiency. Proc. Eng. 83, 365–370. doi: 10.1016/j.proeng.2014.09.029

[B14] Li Y. C. Li Z. Li Z. W. Jiang Y. H. Weng B. Q. Lin W. X. (2016). Variations of rhizosphere bacterial communities in tea (*Camellia sinensis* L.) continuous cropping soil by high-throughput pyrosequencing approach. J. Appl. Microbiol. 121, 787–799. doi: 10.1111/jam.13225 27377624

[B15] Li R. Y. Liu K. Y. Liang Z. W. Luo H. Wang T. An J. S.. (2022). Unpruning improvement the quality of tea through increasing the levels of amino acids and reducing contents of flavonoids and caffeine. Front. Nutr. 9. doi: 10.3389/fnut.2022.1017693 PMC9558131 36245481

[B16] Li X. Qiao L. Huang Y. P. Li D. C. Xu M. G. Ge T. D.. (2023). Manuring improves soil health by sustaining multifunction at relatively high levels in subtropical area. Agric. Ecosyst. Environ. 353, 108539. doi: 10.1016/j.agee.2023.108539

[B17] Liu M. Y. Burgos A. Ma L. F. Zhang Q. F. Tang D. D. Ruan J. Y. (2017). Lipidomics analysis unravels the effect of nitrogen fertilization on lipid metabolism in tea plant (*Camellia sinensis* L.). BMC Plant Biol. 17, 165. doi: 10.1186/s12870-017-1111-6 29037151 PMC5644128

[B18] Liu N. F. Shen. S. S. Huang L. F. Deng. G. J. Wei. Y. M. Ning. J. M.. (2023). Revelation of volatile contributions in green teas with different aroma types by GC–MS and GC–IMS. Food Res. Int. 169, 112845. doi: 10.1016/j.foodres.2023.112845 37254419

[B19] Liu Y. Tian J. Liu B. Zhuo Z. P. Shi C. Xu R. N.. (2022b). Effects of pruning on mineral nutrients and untargeted metabolites in fresh leaves of *Camellia sinensis* cv. Shuixian. Front. Plant Sci. 13. doi: 10.3389/fpls.2022.1016511 PMC9606708 36311102

[B20] Liu Y. Zhuo Z. P. Tian J. Liu B. Shi C. Xu R. N.. (2022a). Directed accumulation of nitrogen metabolites through processing endows Wuyi Rock Tea with singular qualities. Molecules 27, 3264. doi: 10.3390/molecules27103264 35630739 PMC9147623

[B21] Ma L. F. Yang X. D. Shi Y. Z. Yi X. Y. Ji L. F. Cheng Y.. (2021). Response of tea yield, quality and soil bacterial characteristics to long-term nitrogen fertilization in an eleven-year field experiment. Appl. Soil Ecol. 166, 103976. doi: 10.1016/j.apsoil.2021.103976

[B22] Manter D. K. Delgado J. A. Blackburn H. D. Harmel D. de León A. A. P. Honeycutt C. W. (2017). Why we need a national living soil repository. Proc. Natl. Acad. Sci. U S A. 114, 13587–13590. doi: 10.1073/pnas.1720262115 29279417 PMC5748234

[B23] Manzoor Ma L. F. Ni K. Ruan J. Y. (2024). Influence of organic and inorganic fertilizers on tea growth and quality and soil properties of tea orchards’ top rhizosphere soil. Plants 13, 207. doi: 10.3390/plants13020207 38256759 PMC10820999

[B24] Mishima S. Endo A. Kohyama K. (2010). Nitrogen and phosphate balance on crop production in Japan on national and prefectural scales. Nutr. Cycl Agroecosys. 87, 159–173. doi: 10.1007/s10705-009-9324-1

[B25] Morris S. J. Blackwood C. B. (2023). Soil Microbiology, Ecology and Biochemistry. Fifth edition (Amsterdam: Elsevier). doi: 10.1016/B978-0-12-822941-5.00010-7

[B26] Ni K. Liao W. Y. Yi X. Y. Niu S. Y. MA L. F. Shi Y. Z.. (2019). Fertilization status and reduction potential in tea gardens of China. J. Plant Nutr. Fertilizers 25, 421–432. doi: 10.11674/zwyf.18078

[B27] Peng W. T. Zhang L. D. Zhou Z. Fu C. Chen Z. C. Liao H. (2018). Magnesium promotes root nodulation through facilitation of carbohydrate allocation in soybean. Physiol. Plant 163, 372–385. doi: 10.1111/ppl.12730 29572845

[B28] Piyasena K. N. P. Hettiarachchi L. S. K. (2023). Comparison of tea quality parameters of conventionally and organically grown tea, and effects of fertilizer on tea quality: A mini-review. Food Chem. Adv. 3, 100399. doi: 10.1016/j.focha.2023.100399

[B29] Qiu Z. H. Liao J. M. Chen J. H. Li A. S. Lin M. Y. Liu H. M.. (2024). Comprehensive analysis of fresh tea (*Camellia sinensis* cv. Lingtou Dancong) leaf quality under different nitrogen fertilization regimes. Food Chem. 439, 138127. doi: 10.1016/j.foodchem.2023.138127 38064834

[B30] Raza A. Chen C. Q. Luo L. Asghar M. A. Li L. Shoaib N.. (2024). Combined application of organic and chemical fertilizers improved the catechins and flavonoids biosynthesis involved in tea quality. Scientia Hortic. 337, 113518. doi: 10.1016/j.scienta.2024.113518

[B31] Ruan L. Wang L. Y. Wei K. Cheng H. Li H. L. Shao S. J.. (2019). Comparative analysis of nitrogen spatial heterogeneity responses in low nitrogen susceptible and tolerant tea plants (*Camellia sinensis*). Scientia Hortic. 246, 182–189. doi: 10.1016/j.scienta.2018.10.063

[B32] Saha A. Basak B. B. Gajbhiye N. A. Kalariya K. A. Manivel P. (2019). Sustainable fertilization through co-application of biochar and chemical fertilizers improves yi-eld, quality of *Andrographis paniculata* and soil health. Ind. Crops Prod. 140, 111607. doi: 10.1016/j.indcrop.2019.111607

[B33] Shaji H. Chandran V. Mathew L. (2020). Controlled Release Fertilizers for Sustainable Agriculture (Amsterdam: Elsevier). doi: 10.1016/B978-0-12-819555-0.00013-3

[B34] Shi R. Wang Y. Zhou F. Hussain S. Lei X. Y. Chen E. X.. (2024). Nitrogen fertilizer reduction based on bioorganic fertilizer improves the yield and quality of fresh leaves of alpine tea in summer. Beverage Plant Res. 4, 1–10. doi: 10.48130/bpr-0024-0024

[B35] Sönmez O. Turan V. Kaya C. (2016). The effects of sulfur, cattle, and poultry manure addition on soil phosphorus. Turk J. Agric. For. 40, 536–541. doi: 10.3906/tar-1601-41

[B36] Turan V. (2021). Arbuscular mycorrhizal fungi and pistachio husk biochar combination reduces Ni distribution in mungbean plant and improves plant antioxidants and soil enzymes. Physiol. Plant 173, 418–429. doi: 10.1111/ppl.13490 34235745

[B37] Wang Z. H. Gan S. Sun W. J. Chen Z. D. (2022a). Quality characteristics of Oolong tea products in different regions and the contribution of thirteen phytochemical components to its taste. Horticulturae 8, 278. doi: 10.3390/horticulturae8040278

[B38] Wang Y. Liu Y. Zhuo Z. P. Xue J. P. Xu. R. N. Sun. L. L.. (2022b). Effects of high nitrogen and low phosphorus medium potassium ratios on the yield and quality of Wuyi rock tea. Jounral South. Agric. 53, 391–400. doi: 10.3969/j.issn.2095-1191.2022.02.012

[B39] Wang B. Wang S. Li G. Y. Fu L. B. Chen H. Yin M.. (2025). Reducing nitrogen fertilizer usage coupled with organic substitution improves soil quality and boosts tea yield and quality in tea plantations. J. Sci. Food Agric. 105, 1228–1238. doi: 10.1002/jsfa.13913 39319598

[B40] Wei K. Liu M. Shi Y. Zhang H. Ruan J. Zhang Q.. (2022). Metabolomics reveal that the high application of phosphorus and potassium in tea plantation inhibited amino-acid accumulation but promoted metabolism of flavonoid. Agronomy 12, 1086. doi: 10.3390/agronomy12051086

[B41] Withers E. Hill P. W. Chadwick D. R. Jones D. L. (2020). Use of untargeted metabolomics for assessing soil quality and microbial function. Soil Biol. Biochem. 143, 107758. doi: 10.1016/j.soilbio.2020.107758

[B42] Yan P. Shen C. Fan L. C. Li X. Zhang L. P. Zhang L.. (2018). Tea planting affects soil acidification and nitrogen and phosphorus distribution in soil. Agric. Ecosyst. Environ. 254, 20–25. doi: 10.1016/j.agee.2017.11.015

[B43] Yan P. Wu L. Q. Wang D. H. Fu J. Y. Shen C. Li X.. (2020). Soil acidification in Chinese tea plantations. Sci. Total Environ. 715, 136963. doi: 10.1016/j.scitotenv.2020.136963 32014781

[B44] Yang X. D. Ni K. Shi Y. Z. Yi X. Y. Zhang Q. F. Fang L.. (2018). Effects of long-term nitrogen application on soil acidification and solution chemistry of a tea plantation in China. Agric. Ecosyst. Environ. 252, 74–82. doi: 10.1016/j.agee.2017.10.004

[B45] Yang T. Siddique K. H. M. Liu K. (2020). Cropping systems in agriculture and their impact on soil health-A review. Glob. Ecol. Conserv. 23, e01118. doi: 10.1016/j.gecco.2020

[B46] Ye J. H. Ye Y. Yin J. F. Jin J. Liang Y. R. Liu R. Y.. (2022). Bitterness and astringency of tea leaves and products: Formation mechanism and reducing strategies. Trends Food Sci. Tech. 123, 130–143. doi: 10.1016/j.tifs.2022.02.031

[B47] Zhai X. T. Zhang L. Granvogl M. Ho C. T. Wan X. C. (2022). Flavor of tea (*Camellia sinensis*): A review on odorants and analytical techniques. Compr. Rev. Food Sci. F. 21, 3867–3909. doi: 10.1111/1541-4337.12999 35810334

[B48] Zhang H. Li C. Wei K. Liu M. Shi Y. Yang X.. (2023). The reduction of tea quality caused by irrational phosphate application is associated with anthocyanin metabolism. Beverage Plant Res. 3, 10. doi: 10.48130/BPR-2023-0010

[B49] Zhang M. Y. Riaz M. Zhang L. El-desouki Z. Jiang C. C. (2019). Biochar induces changes to basic soil properties and bacterial communities of different soils to varying degrees at 25 mm rainfall: More effective on acidic soils. Front. Microbiol. 10. doi: 10.3389/fmicb.2019.01321 PMC6582450 31249563

[B50] Zheng Y. Hu Q. Wu Z. Bi W. Chen B. Hao Z.. (2022). Volatile metabolomics and coexpression network analyses provide insight into the formation of the characteristic cultivar aroma of oolong tea (*Camellia sinensis*). Lwt 164, 113666. doi: 10.1016/j.lwt.2022.113666

[B51] Zhou B. Chen Y. Zeng L. Cui Y. Li J. Tang H.. (2022). Soil nutrient deficiency decreases the postharvest quality-related metabolite contents of tea (*Camellia sinensis* (L.) Kuntze) leaves. Food Chem. 377, 132003. doi: 10.1016/j.foodchem.2021.132003 35008025

[B52] Zhou Z. Liu. Y. Zhang. L. M. Xu. R. N. Sun. L. L. Liao H. (2019). Soil nutrient status in Wuyi tea region and its effects on tea quality-related constituents. Sci. Agric. Sinica. 52, 1425–1434. doi: 10.3864/j.issn.0578-1752.2019.08.012

[B53] Zhu J. C. Niu Y. W. Xiao Z. B. (2021). Characterization of the key aroma compounds in Laoshan green teas by application of odour activity value (OAV), gas chromatography-mass spectrometry-olfactometry (GC-MS-O) and comprehensive two-dimensional gas chromatography mass spectrometry (GCxGC-qMS). Food Chem. 339, 128136. doi: 10.1016/j.foodchem.2020.128136 33152893

