# Supplementary figures and images for: Organic fertilizer substitution optimizes aroma metabolites in Wuyi Rock tea
Source: Front Plant Sci. 2025 Jun 18;16:1581120. doi: 10.3389/fpls.2025.1581120 (PMC12213686; doi:10.3389/fpls.2025.1581120)

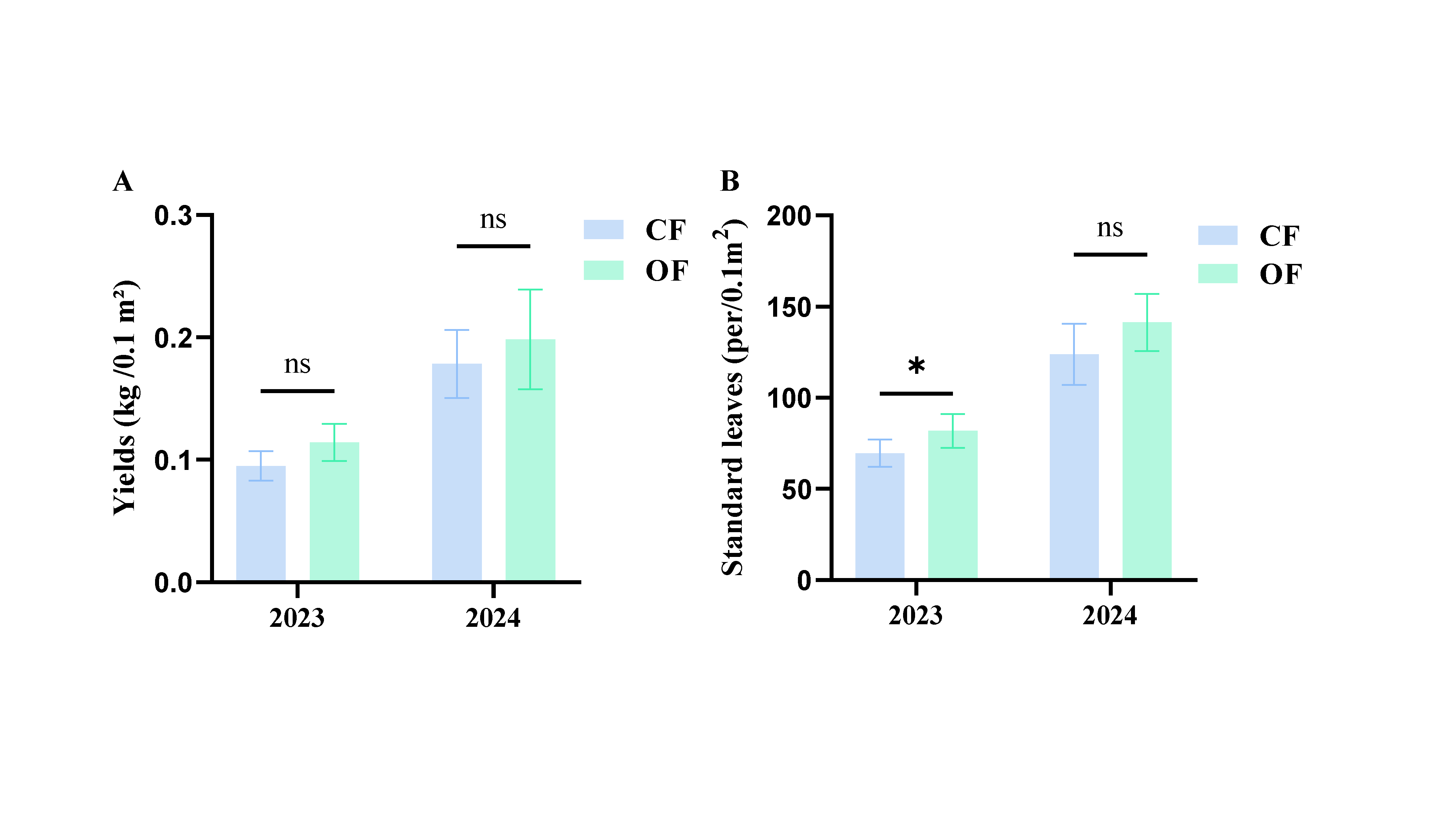

Supplement: Supplementary Figure 1 — Graph representing tea yield under various fertilization treatments. (A) Fresh tea leaf yield within an area of 0.1 m²; (B) Number of standard leaves within an area of 0.1 m². CF, chemical fertilizer; OF, organic fertilizer. Inter-group differences were assessed using t-tests; *0.01 < P ≤ 0.05; **0.001 < P ≤0.01; ***P ≤ 0.001; ns, no significant difference; n = 10. [file Image1.tif]

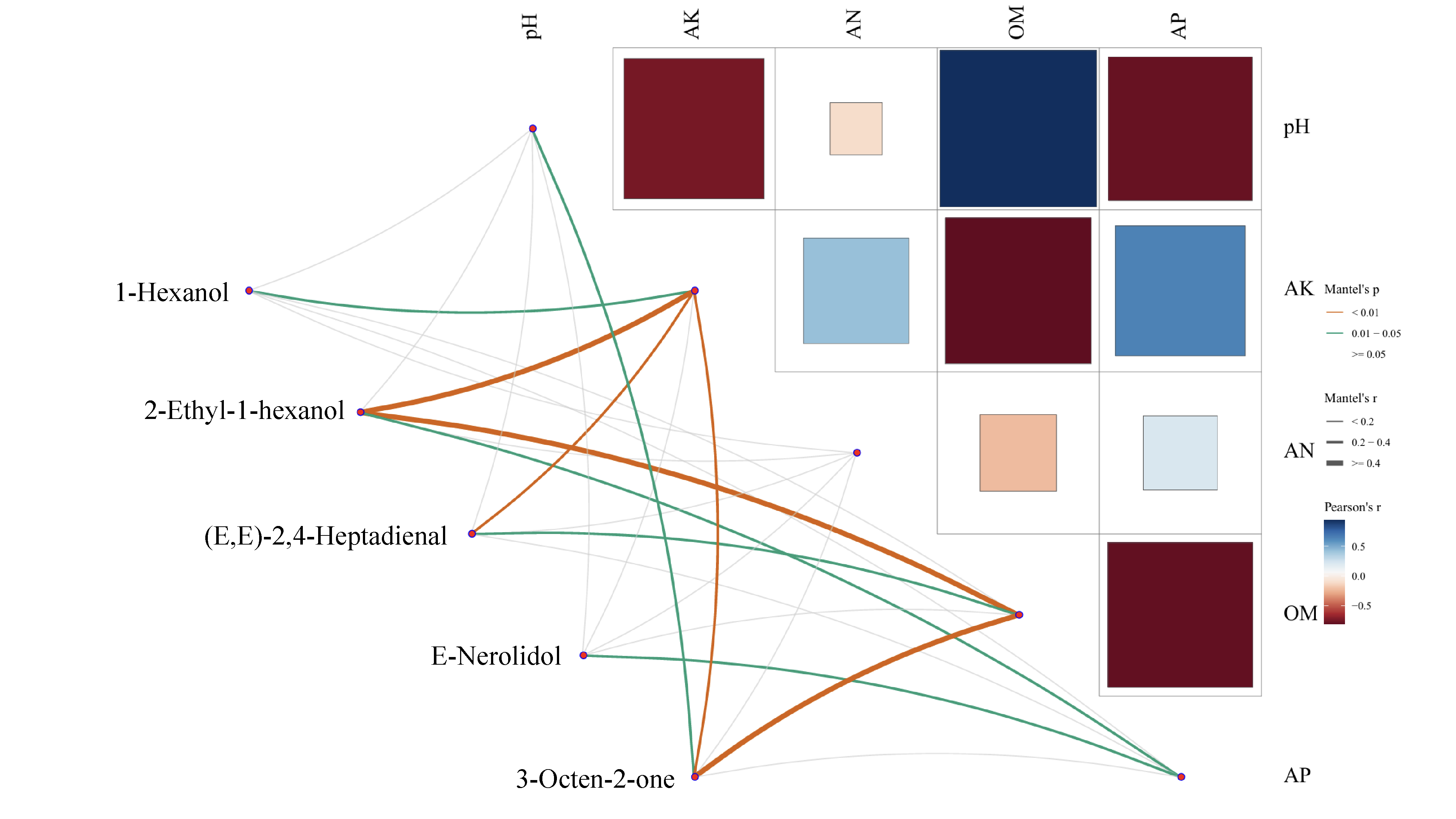

Supplement: Supplementary Figure 2 — Correlation network between soil nutrients and 1-Hexanol, 2-Ethyl-1-hexanol, (E,E)-2,4-Heptadienal, E-Nerolidol and 3-Octen-2-one.The color scale represents Pearson’s correlation coefficients: blue for positive correlations, red for negative correlations. Mantel’s P-values are indicated by light (0.01 to 0.05) and green (> 0.05) shading, with line thickness corresponding to the strength of Mantel’s correlation (r), where thicker lines denote stronger correlations. [file Image2.tif]

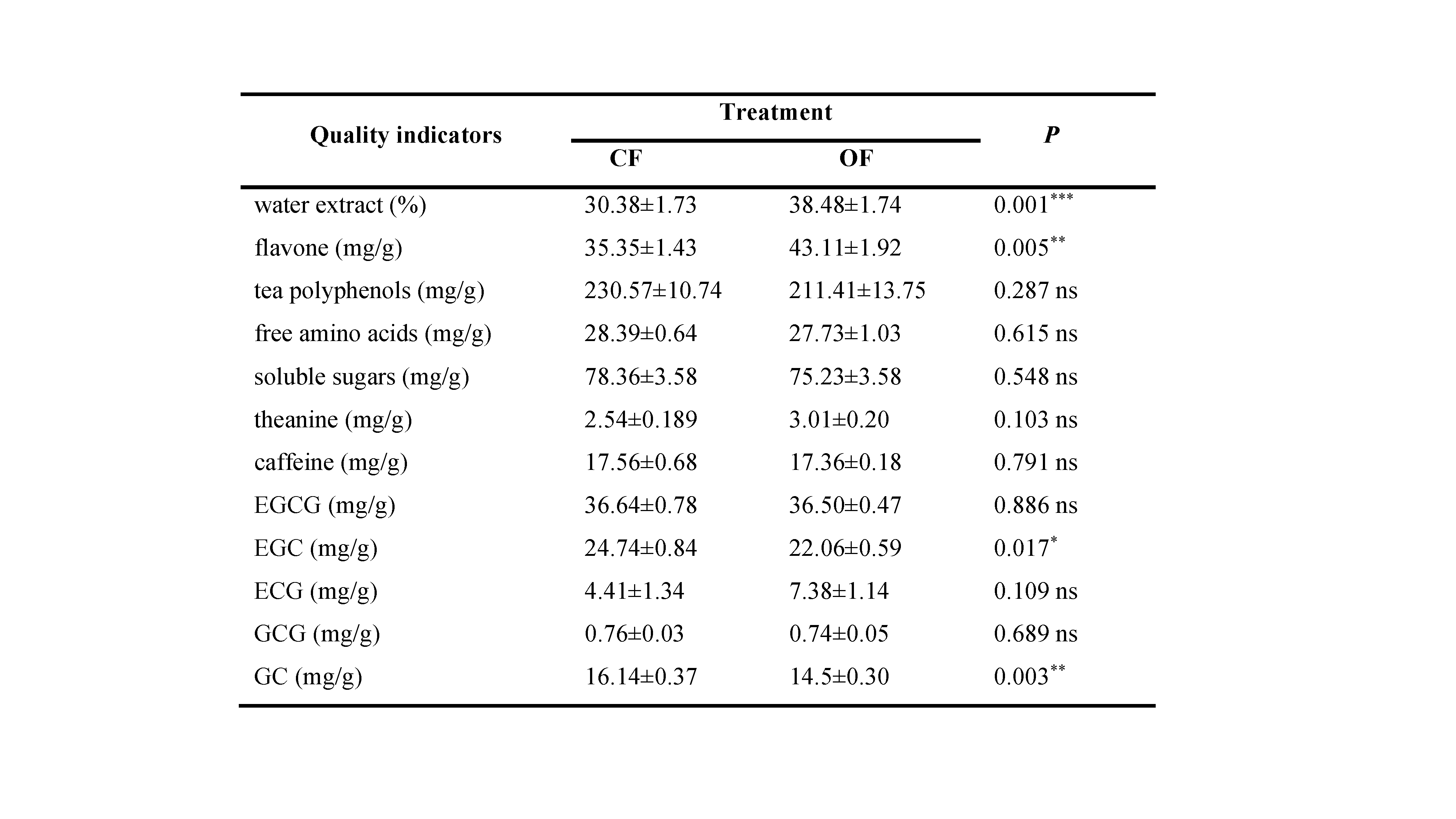

Supplement: Supplementary Table 1 — Concentration of non-volatile metabolites in fresh tea leaves under various fertilization treatments. [file Image3.tif]
